# Expression of *CD 133* in Invasive Ductal Carcinoma of Breast

**DOI:** 10.31557/APJCP.2020.21.10.3055

**Published:** 2020-10

**Authors:** Preeti Ashok Utnal, Hemalatha A, Sreeramulu PN, Manjunath GN

**Affiliations:** 1 *Department of Pathology, Sri Devaraj Urs Medical College, Sri Devaraj Urs Academy of Higher Education and Research, Kolar, Karnataka, India. *; 2 *Department of Surgery, Sri Devaraj Urs Medical College, Sri Devaraj Urs Academy of Higher Education and Research, Kolar, Karnataka, India. *; 3 *Department of Radiooncology, Sri Devaraj Urs Medical College, Sri Devaraj Urs Academy of Higher Education and Research, Kolar, Karnataka, India. *

**Keywords:** Breast carcinoma, cancer stem cells, histopathology, prognosis

## Abstract

**Background:**

CD133 is a commonly used cancer stem cell (CSC) marker in breast cancer. However, the association between *CD133* expression, with clinicopathological features and prognosis in breast cancer, is poorly understood in the Indian subcontinent. This study was designed to explore the expression of *CD 133* in breast carcinoma and to know its association between CD133 and clinicopathological characteristics.

**Methods:**

A total of fifty seven cases were included in the study. All the clinicopathological parameters were collected from Department of Pathology archives. Slides, blocks, clinical information, tumor size and axillary lymph node status were obtained from medical records and the pathology reports. Immunohistochemistry was done using CD 133 antibodies. Both Cytoplasmic and membranous staining was taken a positive. Scoring was done based on percentage of positive cells and intensity of staining. MS Excel, SPSS version 22 (IBM SPSS Statistics, Somers NY, USA) was used to analyze data.p value < 0.05 was considered as statistically significant.

**Results:**

Statistically significant association between the *CD 133* expression and nodal metastasis, tumor stage and Nottingham prognostic index was analysed. There was no statistical correlation between *CD 133* expression age, tumor grade and tumor size. The disease free survival showed the mean disease free survival of CD 133 positivity cases was 16months. And the patients who were negative for *CD 133* expression had mean survival of 30 months. By the Kaplan Mayer graph it was evident that the more the *CD 133* expression the lesser was the disease free survival of the patients.

**Conclusion:**

*CD 133* expression was seen in 77.08% cases and was associated with tumor stage, lymph node metastasis, poor Nottingham prognostic index and worse disease free survival. An increasing trend of association was seen between *CD 133* expression and Age, Tumor Size and Tumor grade.

## Introduction

Breast carcinoma is the most common malignant tumour in women worldwide. In India breast carcinoma is leading cause of malignancy in females with age adjusted rate of 25.8 per 100,000 women and mortality of 12.7 per 100,000 women with higher incidence in Urban area as compared to Rural population (Bray et al., 2018; Malvia et al., 2017). The prognostic and therapeutic outcome in these patients depends on expression of biomarkers such as oestrogen receptor, progesterone receptor, human epidermal growth receptor2 (Her 2 neu) and many others. Inspite of availability of treatment protocols tailored on expression of above markers, relapse and resistance is known to occur. Therefore, more reliable and efficient prognostic markers are required to stratify high-risk population and understand the pathogenesis in such patients.

Amongst newer set of prognostic markers, evidence demonstrates that cancer stem cells (CSCs) play an important role in tumor initiation, occurrence and metastasis. Various markers such as CD34, CD38, CD44, CD133, and ALDH are used to identify these stem cells. CD133, also known as prominin-1, is a trans-membrane glycoprotein expressed in various malignancies, such as breast carcinomas, brain tumors, pancreatic cancer, non-small cell lung cancer, hepatocellular carcinoma, and ovarian cancer. In breast carcinomas, the expression of *CD133* and its relation with histopathological parameters has yielded conflicting results which may be attributed to different research methodologies and differences between study populations (Mukhopadhyay et al., 2013; Zhan et al., 2017; Kim et al., 2015; Margaret et al., 2013). In this regard few studies have been done to look into the role of CD133 in breast carcinoma.

*Objectives of the study are*

To study the expression of *CD133* in Invasive Ductal Carcinoma (Not Otherwise Specified).

To correlate the expression of *CD133* with tumour size, node metastasis, tumour grade, stage and Nottingham prognostic index.

To study the survival of CD 133 positive and CD 133 negative patients.

## Materials and Methods

Institutional ethical clearance was taken before start of the study. Fifty seven patients who underwent modified radical mastectomy for treatment of invasive ductal carcinoma at tertiary hospital in a rural set up from November 2017 to October 2019 were included in the study. Patients who underwent neoadjuvant chemotherapy and radiotherapy were excluded from the study. Sample size was estimated based on the expression of *CD133* in cancer cells which was reported to be 53.3% with 80% level of confidence with absolute error of 10% (Sahar., et al., 2015).

Paraffin blocks and slides were retrieved from the archives of the department of Pathology. Clinical information, tumor size and axillary lymph node status were obtained from medical records and the pathology reports. All the Hematoxylin and eosin stained slides were screened for histological type, tumour grade, nodal metastasis and appropriate slides were chosen for immunohistochemistry.

Immunohistochemical Examination: Four microns thick tissue sections were cut and sections were floated on coated slides. Immunohistochemistry was done using antibodies against CD133 (Abcam – ab18976) on the principle of peroxidase and antiperoxidase method using diaminobenzidine (DAB) for identification of positive reaction (Dako). Negative and positive controls were run simultaneously.

Interpretation of Data:The slides were screened by two pathologists independently. Cytoplasm and membrane staining was taken as positive. The intensity of positivity the cells with no staining were taken as Negative and score 0 , weak staining was score 1, Moderate staining was scored 2 and strongly stained was scored 3. The percentage of cells were scored as 1 when < 10%, score 2 < 11% – 50 %, score 3 <51% - 75%, score 4 and >75%. The final score was calculated by multiplying intensity of CD 133 staining and the extent of positivity. The final score ranged between 0 to 12%.The score>3 was considered as positive.

*Statistical analysis*

Data was entered into Microsoft excel data sheet and was analyzed using SPSS 22 version software. Categorical data was represented in the form of Frequencies and proportions. Chi-square test was used as test of significance for qualitative data. Continuous data was represented as mean and standard deviation. Independent t test was used as test of significance to identify the mean difference between two quantitative variables. p value (Probability that the result is true) of <0.05 was considered as statistically significant after assuming all the rules of statistical test. 

Statistical software: MS Excel, SPSS version 22 (IBM SPSS Statistics, Somers NY, USA) was used to analyze data.

## Results

The study was done in the time period of November 2017 to October 2019. Total 57 cases were included in the study.

*Patient characteristics*

Out of total 57 cases, the youngest was 28 years old and oldest was 92 years. The average ageof presentation was 55 years. 33/57 (57.9%) were of grade 1,17/57 (29.8%) were of grade 2 and 7/57 (12.3%) were grade 3. Majority of tumors were two to five centimeters in size. And metastasis to nodes was seen in 44/57 cases where axillary lymph node dissection was made. Maximum number of cases belonged to NPI IV (28/57). The demographic and histopathological features are represented in Table 1.

Forty four cases out of Fifty Seven cases (77.18%), showed expression of *CD 133* (Score from 1 to 12).

*CD 133* expression (positive and negative) was correlated with age, tumor grade, size, lymph node metastasis, stage and NPI score. The same has been expressed in table 2.In our study there was no statistically significant correlation between *CD 133* expression and Age, Tumor Size and Tumor grade. However, Increased CD 133 positivity was seen in older females, large tumor size and high tumor grade.Out of 57 cases 33 cases showed lymph node metastasis, 28 /33 cases (85%) showed CD 133 positivity. 24 cases were negative for lymph node metastasis, 11/24 cases (46 %) were positive for *CD 133* expression. The CD 133 positivity and lymph node metastasis was statistically significant (p value - 0.005). *CD 133* expression also correlated with tumor stage and NPI.

Data on Estrogen receptors (ER), Progesterone receptors(PR) and HER 2 neu receptors (immunohistochemistry) expression and *CD 133 *expression was obtained in six cases. Two patients were triple negative, with *CD 133* expression of 9 and 12. In, other four patients two patients belonged to Luminal A, one patient had Luminal B and another one belonged to Luminal C type of breast carcinoma. The number of cases with ER, PR, and Her 2neu data were few for statistical analysis.

Out of 57 cases follow up was done in 33 cases for a period of 8 months to 41 months after the surgery. There was no distant metastasis during the surgery in any of the cases. Out of these 33 cases 3 cases expired during the follow up due to causes unrelated with the disease process. Out of these 33 cases 21 cases showed CD 133 positivity with a mean survival of only 16 months and 12 cases showed CD133 negativity with the mean survival of 30 months. The graphical representation of same is in the Figure 3 and Figure 4.

**Figure 1:**
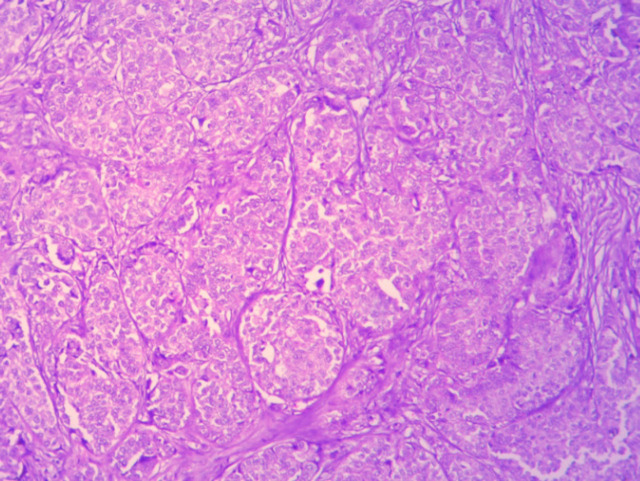
Invasive Ductal Carcinoma Breast (NOS) – H & E – 100x

**Figure 2:**
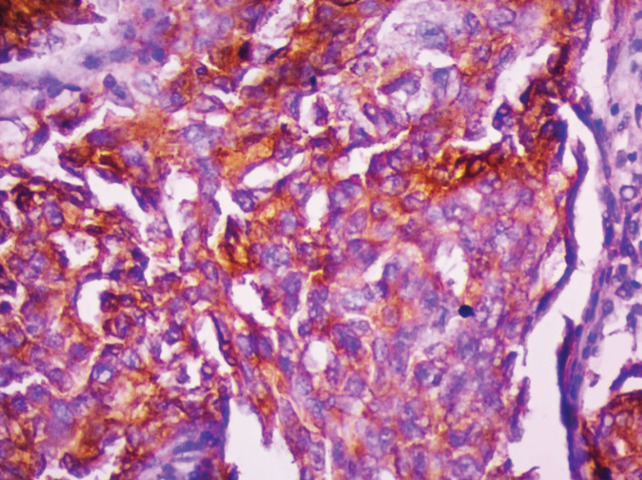
Invasive Ductal Carcinoma - CD 133 IHC – 100x (Extent of positivity >75%- Score 4)

**Figure 3:**
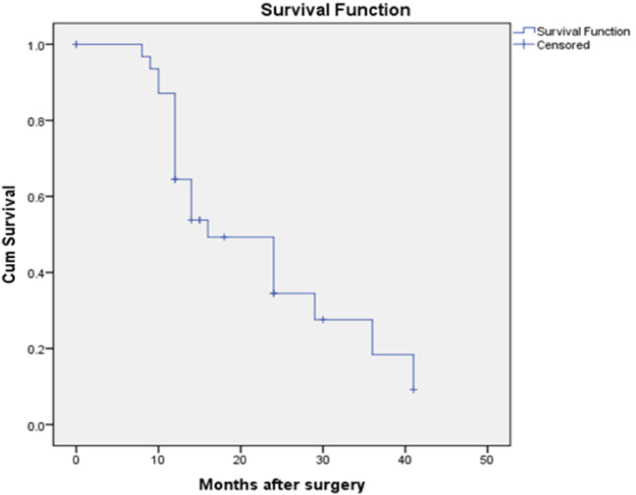
Correlation between CD133 Expression and Disease Free Survival in CD 133 Positive Cases

**Figure 4:**
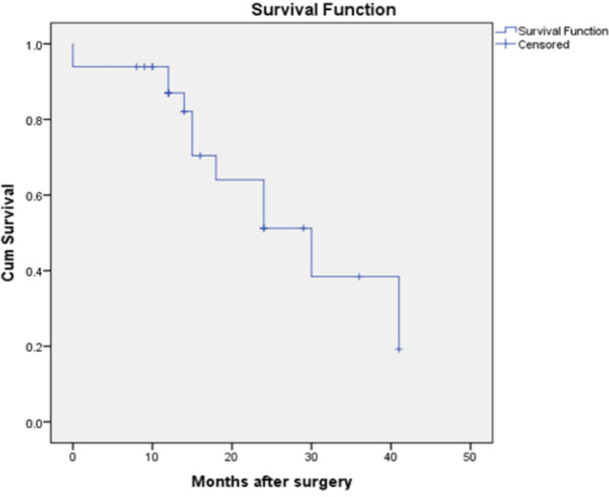
Correlation between CD133 Expression and Disease Free Survival in CD 133 Negative Cases

**Table 1:** Distribution of Cases into Histopathological Parameters

Histopathological Parameters	Number of cases (n = 57)	Percentage (%)
Age		
21-40	15	26.30%
41- 60	31	54.40%
61-80	9	15.80%
81-100	2	3.50%
Grade of the tumor		
I	33	57.90%
II	17	29.80%
III	7	12.30%
Tumor size		
T1 (<2 cms)	8	14.00%
T2 ( 2 - 5 cms)	23	40.40%
T 3 (>5 cms)	19	33.30%
T4 (chest wall)	7	12.30%
Lymph nodes		
Stage N0 (0)	13	22.80%
Stage N1 (1-3)	18	31.60%
Stage N2 (4-9)	7	12.30%
Stage N3 (>9)	19	33.30%
Nottingham Prognostic Index		
I	1	1.80%
II	16	28.00%
III	28	49.10%
IV	12	21.10%

**Table 2:** Correlation of CD133 with Different Histopathological Parameters

Histopathological parameters	CD 133 (n = 57)		P- value
	Positive	Negative	
Age			
< 60years	31	15	0.505
> 60years	8	4	
Tumor grade			
I	20	12	
II	13	5	0.447
III	6	1	
Tumor size			
1(<2cms)	5 (62.5%)	3 (37.5%)	0.218
2(2-5 cms)	12 (52.17%)	11 (47.83%)	
3(>5cms)	16 (82.21%)	3 (15.79%)	
4(Chest wall)	6 (85.71%)	1 (14.29%)	
Lymph node			
N0(0)	11	13	
N1(1-3)	17	3	0.005*
N2(4-9)	7	1	
N3(>9)	4	1	
Tumor stage			
I	2	1	
II	13	14	0.006*
III	24	3	
NPI			
I	0	1	
II	5	11	0.001*
III	23	5	
IV	11	1	

## Discussion

Stem cells play a very important role in the field of malignant tumor biology and have become the topic of debate since its fundamental theory was put forward. Since then, it has been considered that the tumor is composed of tumor cells and CSCs - rare subpopulation of cells in solid tumors with the capability of self-renewal, differentiation potential and initiating tumors (Reya et al., 2001).

Cancer Stem Cells were first discovered by eminent German biologist Ernst Haeckel. Initially CD 133 was found in hematopoietic stem cells and was considered as specific molecular biomarker for hematopoietic stem cells, later its role as a stem cell marker has been proven. .In breast carcinoma meta-analysis show that the CD 133 has been associated with high grade of the tumor, high tumor stage, poor prognosis, poor disease free survival rate and overall bad prognosis (Spangrude, 1988)

In the present study we have observed the expression of* CD 133* in Infiltrating Ductal Carcinoma of Breast and compared the positive expression of *CD 133* expression with age, grade, stage, lymph node metastasis, NPI and DFS of the patient. In the present study, the age group ranged from 28 years to 80 years with mean age of 54 years, which is similar to the other study (Margaret et al., 2013). Though 44% of cases were aged less than 50 years and 56 % cases were above 50 years, statistical significance was not seen with respect to *CD 133 *expression in pre and postmenopausal ages in our study even though some studies have described the association of *CD 133* expression in cancers of Post-menopausal age group (Margaret et al., 2013; Anwar, 2019)

In present study the frequency (score > 1) of *CD 133* expression was seen in 77.19 % of cases (44/57). However a significant expression (score >3) was seen in 39/57 (68%) of cases. This finding of frequency of expression was consistent with studies where frequency of expression of 77.5% (31/40)10 and 87% (67/77) has been reported. (Liou, 2018) In contrary some studies have described expression as low as (22/89), 24.74% (72/291), and 40%-50% (Kim, 2015; Margaret, 2013; Maeda et al., 2008; Han et al., 2015) This wide range of expression may be due to changes in geographical distribution and genetic makeup of the cases. However most of the Indian studies have described a range similar to our study.CD 133 was expressed more in Grade 2 and 3 cases as compared to Grade 1 cases. However the comparison was not statistically significant. A trend of higher incidence of *CD133* expression was noted with advanced histology grade which was consistent with result obtained in other studies (Anwar et al., 2019; Liou et al., 2019; Han, 2015). This may be due to availability of less number of cases in our study when compared with other studies.

Large tumors were associated with increased score of *CD133* expression. However the findings did not reach any statistical significance. The present study showed highest number of cases belonging to T2 i.e 23 cases (40.35%) which correlated with other studies (Anwar, 2019). In present study there was no correlation between the tumor size and the *CD 133* expression. Similarly other studies have found no association between the tumor size and *CD 133* expression (Kim, 2015; Margaret et al., 2013; Mansour, 2015). In contrary some studies have found significant correlation proving that tumor cell turnover has significant relationship with the *CD 133* expression.(Joshi, 2018).

However when correlating with the *CD 133* expression and large tumor size (>5 centimeters and chest involvement – T3 and T4) a significant statistical correlation was observed, these findings were similar to findings in other studies (Kim, 2015; Margaret et al., 2013; Joshi, 2018). This shows that CD 133 correlates with the higher tumor size of infiltrating ductal carcinoma of Breast. More studies needs to be done to see if CD 133 positive cells increases the cell turnover or large tumors acquire increased expression of *CD133* expressing cells.

Our findings of CD 133 and lymph node metastasis was statistically significant which correlated with other studies (Kim, 2015; Liou, 2019; Liu, 2009). However some of the study did not show any correlation between *CD 133* expression and lymph node metastasis (Collina, 2015). *CD 133* expression is important for tumor to spread along the lymphatic channels by the process of epithelial mesenchymal transition hence increased expression helps the cells to invade the lymphatics and spread through lymphatics channels.

Increase in the tumor stage correlated with the *CD 133 *expression. The higher the stage the more was the *CD 133 *expression. The similar findings were seen in the study (Kim , 2015; Margaret et al., 2013; Mansour, 2015). This proves the aggressive nature of the tumor that makes the tumor metastasize and CD 133 positive cells may play an important role in developing metastatic potential.

Nottingham prognostic index, is a well-established prognostic factor calculated based on tumor size, lymph node involvement and tumor grade. It serves as an indirect evidence of prognosis and helps to predict the disease free survival of the breast carcinoma cases. In our study, though the tumor size and grade did not correlate with *CD 133* expression, the totality, comprising of tumor size, lymph node involvement and tumor grade in the form of Nottingham prognostic index correlated with* CD 133 *expression. However many studies have not looked into the association between *NPI* and *CD133* expression.

CD 133 is known to be associated with triple negative breast carcinoma. In our study we had data of *ER, PR* and *Her 2 neu* expression in six cases. Two out of six were Triple negative. However the number of cases with data on ER, PR , Her 2 neu were too less to arrive at statistical conclusion. Literature search revealed that indeed CD 133 is increasingly expressed in triple negative cases, further nuclear localization of CD 133 was more often observed in such cases along with the membranous and cytoplasmic expression. More studies needs to be done in this regard (Collina., 2015). We also related the expression of *CD 133* with disease free survival. Out of 57 cases we could follow up 33 patients. Out of 33 patients, 21 cases showed CD 133 positivity with a mean disease free survival of 16 months. Twelve patients who were negative for *CD 133* expression had mean survival of 30 months. By the Kaplan Miere graph it is evident that the more the *CD 133* expression the lesser was the disease free survival of the patients. Our findings were similar to a meta-analysis involving four studies which showed that increased *CD133* expression in breast cancer patients had poorer Disease Free Survival. They also interpreted that on subgroup analysis according to region and sample size, there was a different trend between the Asian and non-Asian groups, wherein patients in the Asian group with tumors that showed high expression of* CD133* tended to have a poorer overall survival (Zhan et al., 2017).

In conclusion, CD133 markers may potentially serve as prognostic markers and novel potential therapeutic targets in breast cancer. Many studies are also being conducted on using targeted therapy for CD 133 with promising results. However similar studies with more number of samples are required to study *CD 133* expression in molecular subtypes of breast carcinoma.

s
